# Decarbonisation of eastern European economies: monitoring, economic, social and security concerns

**DOI:** 10.1186/s13705-022-00342-8

**Published:** 2022-03-21

**Authors:** Mirjana Radovanović, Sanja Filipović, Simonida Vukadinović, Milovan Trbojević, Iztok Podbregar

**Affiliations:** 1 Faculty of Security Studies, Educons University, Vojvode Putnika 87, Sremska Kamenica, 21208 Republic of Serbia; 2 Institute of Social Sciences, Singidunum University, Danijelova 32, Belgrade, 11000 Republic of Serbia; 3 Department of Economics and Finance, Faculty of Business Economics, Educons University, Vojvode Putnika 87, Sremska Kamenica, Republic of Serbia; 4 Department of Organization and Management, Faculty of Organizational Sciences, University of Maribor, Kidričeva cesta 55a, Kranj, Slovenia

**Keywords:** Decarbonisation, Eastern European economies, Monitoring, Social implications, Security aspects

## Abstract

**Background:**

Decarbonisation of the European economy is one of the main strategic goals of energy transition in the European Union (EU), which aims to become a leader in this process by 2050 and to include other European countries making thus the European continent the first carbon neutral region in the world. Although decarbonisation is an important goal of the EU, the models for monitoring the progress of this process have not yet been clearly defined, and views on the social, economic, and security implications in terms of prioritising decarbonisation are conflicting. The main objective of this paper is to determine the methodological correctness of the existing method of decarbonisation monitoring, to develop a new monitoring model indicating the differences in the EU and European countries that are non-EU and to point out the underlying social, economic and security implications that must certainly find their place in the decision-making process in this field.

**Results:**

The main results showed that there is no clearly defined model for monitoring the success of decarbonisation, while the indicators that are commonly used for this purpose make a model that, as the analysis shows—is methodologically incorrect. In the case of EU countries, the following indicators proved to be the most reliable: *consumption-based CO*_*2*_ and *share in global CO*_*2*_. For non-EU countries, the best monitoring indicators are *CO*_*2*_* per unit of GDP*, *consumption-based CO*_*2*_, and *renewable energy consumption*. These indicators can explain 99% of the variance in decarbonisation success.

**Conclusions:**

The basic conclusion of the paper is that even before the implementation, the decarbonisation monitoring model should be defined and methodologically tested, and the use of a single model for all EU countries or for all countries is not recommended. It is proposed to simplify the monitoring model, with an emphasis on monitoring of *consumption-based CO*_*2*_, which proved to be the most efficient in all sampled countries. The current method of monitoring is based exclusively on environmentally related indicators while ignoring the fact that decarbonisation is associated with almost all aspects of development. The additional social, economic and security aspects need to be developed and included in the further monitoring process.

## Background

The European Union (EU) adopted *Climate Law* [1], and thus committed to reducing carbon emissions by 55% compared to 1990 by 2030, i.e., that Europe will become climate neutral by 2050, which is in line with the previously adopted strategic document *European Green Deal*. The EU member states must harmonise their national legislations with *European Climate Law* by September 2023 and define their own decarbonisation strategies. In other words, all EU countries, as well as the EU accession countries, will have to switch from fossil fuels to 94–96% carbon-free power, which is possible with electrification based on renewable energy sources, with an emphasis on increasing energy efficiency, increased use of electric transport systems and heat pumps, and the production of hydrogen and synthetic fuels [2]. In order to achieve climate neutrality of Europe, it is necessary to successfully implement the energy transition in neighbouring countries, which are part of Europe, but in which the energy transition process is slowed down for a number of reasons [3].

For the purposes of analysis and methodological evaluation of indicators that most often monitor the decarbonisation process, a sample of nine countries (Russian Federation, Moldova, Estonia, Latvia, Lithuania, Armenia, Azerbaijan, Georgia and Ukraine) was selected in this paper. They were once part of the USSR, while today the three Baltic countries are the EU member states. The sample was designed so that based on the analysis of 17 selected indicators for a longer period of time 1990–2019, it could be concluded whether the existing measurement of decarbonisation is possible, i.e., whether it is methodologically correct depending on whether a country is part of the EU.

The Russian Federation, as one of the largest emitters of greenhouse gases in the world, is implementing the transition process slowly because it traditionally relies on fossil fuels and generates significant revenues from their exports [4]. The first actual step towards decarbonisation was the presidential Decree *On the reduction of greenhouse gas emissions* (2013), with a plan to reduce emissions to the level of 75% by 2020, compared to 1990 levels, which was frowned upon by fossil fuels exporters. In March 2020, a draft *Strategy for the long-term development of Russia with a low level of greenhouse gas emissions until 2050* was adopted, but the results of the implementation of carbon mitigation are not transparent. Relations between the Russian Federation and the EU on energy issues are highly dependent, but the Russian Federation has not harmonised its energy transition with the EU's long-term plans, as it exclusively plans to increase the intensity of oil and natural gas exports, with diversification of export markets [5]. Specific resistance to decarbonisation comes from the coal-producing sector, which is supported by the government that plans to expand coal exploitation capacity [6]. The Russian Federation is developing its climate policy in line with the global concept, but key issues of climate policy are still not fully integrated into national social, environmental and economic policies [7].

The Russian Federation emphasises that the export of energy Is nothing but trade, while the European Union, and especially the United States, believe that the Russian Federation uses energy for political purposes. With the transition of the European Union to decarbonisation, and bearing in mind that natural gas is an environmentally friendly energy source, additional security tensions can be expected in this regard. Olga Khrushcheva & Tomas Maltby (2016) The Future of EU-Russia Energy Relations in the Context of Decarbonisation, Geopolitics, 21:4, 799–830, https://doi.org/10.1080/14650045.2016.1188081. The impact of COVID-19 pandemic, rising energy prices and disagreements over Nord Stream 2, further complicate relations between all stakeholders, so the security issues take precedence in the relations of all stakeholders, with possible consequences on the global level [8].

Moldova, a country with low GDP and moderate energy resources, can be characterised as critical regarding energy transition in any respect, with a theoretical approach to decarbonisation [9]. Moldova is a country poor in natural resources, with a very weak economy, completely dependent on energy imports, and thus on the policy of the Russian Federation [10].

Armenia is highly dependent on energy imports from the Russian Federation, as only 35% of its energy is generated from its own sources, with one existing and operational nuclear reactor. There are efforts so Armenia is more actively engaged in climate change activities, which are currently in the form of proposed strategic solutions [11]. Armenia, primarily due to the development of the economy and energy security, has largely given up on the EU path and has adhered to the policy of the Russian Federation. Therefore, it can be said that future security issues pertaining to Armenia will be largely related to the policy of the Russian Federation [12].

Azerbaijan produces four times the energy it consumes, where revenues from oil and natural gas exports account for 90% of total export revenues. As much as 90% of electricity is obtained from natural gas, while the share of renewable energy is only 2% [13]. For now, it seems that there is still no readiness for the energy transition process [14]. Owing to its wealth of energy resources and adequate management system, Azerbaijan is a country that can create its own policy with a greater degree of independence. Significant security issues related to energy exports and decarbonisation are unlikely to be expected [15]. However, the fact that the USA has had a strong influence on Azerbaijan since the collapse of the USSR should be taken into account when observing the energy transition of this country [16].

Georgia ratified the *Kyoto Protocol* and signed a special *EU–Georgia Association Agreement* (2016), which emphasises the need for cooperation in the field of climate change, emission trading, changes in industry and development of clean technologies. Furthermore, as part of the *USAID-funded initiative Institutionalization of Climate Change Adaptation and Mitigation*, Georgia prepared *The Georgian Road Map on Climate Change Adaptation*, with clearly defined priorities. Regardless of good and adequately prepared strategies, implementation is very slow, primarily due to inadequate institutional capacity at all levels [17]. Georgia produces 35% of its energy from its own sources, relying almost entirely on oil and natural gas imports, mostly from the Russian Federation, while the transition to the economy market is at initial phase [18]. However, insufficient access to the scientific literature and poor database are certainly a problem for more accurate assessments of the process of energy transition and decarbonisation in this country [19].

The European Union seeks to exert influence in the South Caucasus region, including Georgia. However, despite numerous efforts and the existence of the *European Union Monitoring Missio*n, there are no significant developments in the field of defense and security policy. Georgia relies on the Russian Federation and its foreign policy in many spheres, so the decarbonisation will most likely occur accordingly [20].

Ukraine has adopted a number of policies aimed at improving its energy security, but remains highly dependent on energy imports, with no significant progress in the field of energy efficiency and climate change mitigation. Ukraine produces electricity in coal-fired, nuclear and hydropower plants, but the infrastructure is extremely outdated [21]. The energy product market is still state-owned with a high degree of corruption [22]. Ukraine intends to join the EU and implement decarbonisation, but is facing great efforts to align its policies and show readiness for change, so the actual results in the field of decarbonisation are missing [23].

The example of Ukraine probably best reflects the complexity and interconnection of energy security, decarbonisation and politics, with insufficiently transparent positions of the Russian Federation, the European Union and the United States. Ukraine itself is also facing division, i.e., vaguely defined future and the primary partner (the European Union or the Russian Federation). Tensions have also led to the militarisation of the region, which certainly calls into question the essential stability and development of this country, as well as decarbonisation. No progress can be expected in a tense security situation [24].

Estonia, as an EU member state, is obliged to implement EU strategies and directives, so net zero by 2050 is the goal that Estonia should achieve and incorporate into its policies. However, so far there have been difficulties in implementing the energy transition, as the issue of security and defence is strongly integrated into energy policies. The USA is an important ally of Estonia, but changes in foreign policy under President Donald Trump have led to a major stalemate in energy and climate policies. Estonia certainly wants to maintain strong ties with the USA (for security reasons), but this leads to a conflict when it comes to obligations towards the EU in certain areas, including climate change [25]. Estonia is one of the three leading EU countries in terms of greenhouse gas emissions per capita, due to rapid economic growth, the use of energy-intensive shale-based energy, increased traffic intensity and high building energy consumption [26]. Given the stated political and economic reasons, the status of the decarbonisation process in Estonia cannot be adequately assessed, which is evident from the small volume of scientifically based literature and research.

The influence of the great powers is exerted through the influence of the European Union and the Russian Federation, with the increased influence of China (through investment, primarily in the energy sector and decarbonisation). Given that Estonia is part of the European Union, these activities may have the opposite effect: good results in decarbonisation, but increased Chinese influence in this country [27].

Latvia traditionally relies on energy production using oil shale and hydropower [28], and the implementation of the first measures aimed at energy transition was ineffective [29]. Energy intensive industry is a large part of the structure of Latvian industry, which receives substantial financial subsidies [30]. Naturally, the assessment of energy performance and decarbonisation processes is difficult due to no reliable data [31].

Before the crisis in Ukraine (2014), Latvia was largely oriented towards the European Union. However, after 2014, it has become noticeable that all development plans, as well as security, put the Russian Federation in the forefront as a partner of interest. Given Latvia’s high degree of dependence on energy imports from the Russian Federation, it can be expected that future success of decarbonisation will largely depend on mutual relations between the two countries, especially in terms of security [32].

Lithuania has significantly reduced energy intensity, but the energy transition is certainly hampered by the fact that the government significantly subsidises the prices of natural gas and electricity, which are twice lower than the EU average [33]. On the other hand, studies show that the largest contribution to decarbonisation in Lithuania can be expected from the production of energy from renewable sources [34].

Given that Lithuania imports about 70% of its electricity (as crucial for decarbonisation) from the Russian Federation, it can be rightly argued that the success of the country’s energy transition will largely depend on relations with the Russian Federation [35]. The situation can further be complicated by the fact that Lithuania is a member of the European Union and NATO. The different interests of the Russian Federation and NATO can theoretically lead to destabilisation and problems in the decarbonisation of this country. Since security, energy and decarbonisation are closely linked, further developments are yet to come [36].

The energy transition in the EU has led to some disagreements among its member states on how to implement it and on the established priorities, and therefore two approaches have emerged: countries that have embraced the shift to renewables, improved energy efficiency and active reduction of carbon emissions, and on the other hand, countries that have prioritised energy security and stability of supply, which certainly has its political and geopolitical implications [37]. It can be expected that an ambitious strategy for the complete decarbonisation of the EU (with plans for neighbouring countries) by 2050 will lead to even greater differences among European member states. Properly determining the success of decarbonisation and the model for its monitoring that has passed the methodological verification are imposed as one of the key problems, because they make important and long-term decisions based on monitoring [38]. The subject of this paper is the assessment of ways to monitor decarbonisation in two groups of European countries (EU countries and non-EU countries), as well as the assessment of the impact of EU membership on changes in this area, assessment of methodological correctness of the previous model and proposal of further monitoring model.

## Methods

Research sample includes countries at the lower (or unknown) level of the carbon mitigation: Russian Federation, Moldova, Estonia, Latvia, Lithuania, Armenia, Azerbaijan, Georgia and Ukraine. The energy sector in selected countries (all countries were members of former USSR) was characterised by a high level of energy intensity, high energy consuming in industry sector, in certain countries high exploitation of fossil fuels and a high energy-related pollution in general. The energy transition has started in these countries as well (three countries in the sample have become members of the EU), but with an unclear effect. The research covers the period 1990–2019 and includes 12 selected CO_2_-related indicators and 5 other indicators. A set of statistical tests will be applied in the paper to assess the methodological correctness of monitoring the process of decarbonisation in nine former USSR countries, three of which are now EU members (Estonia, Latvia and Lithuania).

All these countries share a similar industry mix (energy-intensive industry), high dependence on fossil fuels (either own or imported), low level of energy production from renewable sources, high energy consumption per unit of GDP, and are slow to implement climate change policies in general [39]. The indicators were selected in line with the objective of the paper:

1. CO_2_ per capita
2. CO_2_ emission (total)
3. Year-to-year CO_2_ change
4. Cumulative CO_2_ emission
5. CO_2_ consumption based
6. CO_2_ share
7. CO_2_ emission (cement)
8. CO_2_ emission (coal)
9. CO_2_ emission (flaring)
10. CO_2_ emission (gas)
11. CO_2_ emission (oil)
12. CO_2_ emission (other)
13. GDP change—auxiliary indicator
14. GDP per capita—auxiliary indicator
15. Renewable energy consumption—auxiliary indicator
16. Renewable electricity output—auxiliary indicator
17. CO_2_ per unit of GDP—as an assumed basic indicator of decarbonisation success

Indicators directly related to CO_2_ (from 1 to 12) were used in the first phase of the research, while indicators from 13 to 17 were used in the second phase. Indicator *CO*_*2*_* per unit of GDP* was used in all phases of the analysis.

## Results

The research was conducted in two basic stages. An overall assessment of the methodological correctness of the selected group of indicators for monitoring the success of decarbonisation was made in the first stage of the research, while the differences between EU countries and non-EU countries were assessed in the second stage.

### First research stage—basic methodological assessment

The correlations between individual indicators were determined in the first phase of the paper, by applying *Pearson's correlation coefficient*, and the results are shown in Table 1.

**Table 1:** Correlations of decarbonisation indicators—*Pearson’s correlation coefficient*

	CO_2_ per capita	CO_2_ emission	Year-to-year CO_2_	Cumulative CO_2_	Consumption CO_2_	Share of CO_2_	Cement CO_2_	Coal CO_2_	Flaring CO_2_	Gas CO_2_	Oil CO_2_	Other CO_2_
CO_2_ per capita	1	0.530^**^	− 0.129^*^	0.491^**^	0.265^**^	0.528^**^	0.521^**^	0.546^**^	0.401^**^	0.505^**^	0.521^**^	0.428^**^
CO_2_ emission	0.530^**^	1	− 0.211^**^	0.958^**^	0.993^**^	0.981^**^	0.979^**^	0.987^**^	0.926^**^	0.989^**^	0.975^**^	0.972^**^
Year-to-year CO_2_	− 0.129^*^	− 0.211^**^	1	− 0.137^*^	− 0.521^**^	− 0.280^**^	− 0.176^**^	− 0.223^**^	− 0.046	− 0.185^**^	− 0.238^**^	− 0.259^**^
Cumulative CO_2_	0.491^**^	0.958^**^	− 0.137^*^	1	0.917^**^	0.896^**^	0.942^**^	0.940^**^	0.953^**^	0.979^**^	0.881^**^	0.871^**^
Consumption CO_2_	0.265^**^	0.993^**^	− 0.521^**^	0.917^**^	1	0.966^**^	0.954^**^	0.987^**^	0.712^**^	0.978^**^	0.893^**^	0.984^**^
Share of CO_2_	0.528^**^	0.981^**^	− 0.280^**^	0.896^**^	0.966^**^	1	0.942^**^	0.981^**^	0.843^**^	0.952^**^	0.980^**^	0.978^**^
Cement CO_2_	0.521^**^	0.979^**^	− 0.176^**^	0.942^**^	0.954^**^	0.942^**^	1	0.959^**^	0.948^**^	0.969^**^	0.956^**^	0.959^**^
Coal CO_2_	0.546^**^	0.987^**^	− 0.223^**^	0.940^**^	0.987^**^	0.981^**^	0.959^**^	1	0.877^**^	0.963^**^	0.959^**^	0.967^**^
Flaring CO_2_	0.401^**^	0.926^**^	− 0.046	0.953^**^	0.712^**^	0.843^**^	0.948^**^	0.877^**^	1	0.956^**^	0.861^**^	0.856^**^
Gas CO_2_	0.505^**^	0.989^**^	− 0.185^**^	0.979^**^	0.978^**^	0.952^**^	0.969^**^	0.963^**^	0.956^**^	1	0.938^**^	0.929^**^
Oil CO_2_	0.521^**^	0.975^**^	− 0.238^**^	0.881^**^	0.893^**^	0.980^**^	0.956^**^	0.959^**^	0.861^**^	0.938^**^	1	0.991^**^
Other CO_2_	0.428^**^	0.972^**^	− 0.259^**^	0.871^**^	0.984^**^	0.978^**^	0.959^**^	0.967^**^	0.856^**^	0.929^**^	0.991^**^	1

^**^Correlation is significant at the 0.01 level (2-tailed)

^*^Correlation is significant at the 0.05 level (2-tailed)

The results in Table 1 show that all indicators have high correlations (at the level of *p* < 0.05 or *p* < 0.01). It is already evident at this point that the monitoring model that would include all the above indicators is not methodologically correct, because high correlations clearly indicate a poorly established model, so further testing is required.

*CO*_*2*_* per unit of GDP* has been identified as one of the basic indicators of the success of the energy transition, therefore additional attention is paid to observing this indicator in relation to others. This indicator is positively correlated with all other indicators, except the indicator *year-to-year CO*_*2*_* change*, where the correlation is negative, while there is no statistically significant correlation with *flaring CO*_*2*_.

Given the above, two opposing conclusions can be drawn. First, the indicator *CO*_*2*_* per unit of GDP* can be used as the only indicator to assess the success of energy transition or this indicator does not contribute to the tested model. In order to test the hypothesis on these two ways of making a conclusion, an exploratory method was used to estimate the correlation of *CO*_*2*_* per unit of GDP* with other indicators, and the results are shown in Table 2.

**Table 2:** Correlations between *CO*_*2*_* per unit of GDP* and selected decarbonisation indicators—*Pearson’s correlation coefficient*

		CO_2_ per capita	CO_2_ emission	Year-to-year CO_2_	Cumulative CO_2_	Consumption CO_2_	Share of CO_2_	Cement CO_2_	Coal CO_2_	Flaring CO_2_	Gas CO_2_	Oil CO_2_	Other CO_2_
CO_2_ per unit of GDP		0.387^**^	0.187^**^	− 0.178^**^	0.163^**^	0.502^**^	0.223^**^	0.139^*^	0.244^**^	0.076	0.160^**^	0.168^**^	0.233^**^

^**^Correlation is significant at the 0.01 level (2-tailed)

^*^Correlation is significant at the 0.05 level (2-tailed)

Table 3 shows a regression model, which aims to assess the share of individual indicators in explaining the presented model of monitoring the success of decarbonisation.

**Table 3:** Summarised results of the regression model of selected decarbonisation indicators

Model	*R*	*R* square	Adjusted *R* square	*R* square change
1	0.993^a^	0.987	0.987	0.987
**2**	**0.999**^**b**^	**0.997**	**0.997**	**0.010**
3	0.999^c^	0.999	0.999	0.001
4	1.000^d^	0.999	0.999	0.001
5	1.000^e^	0.999	0.999	0.000
6	1.000^f^	1.000	1.000	0.000
7	1.000^g^	1.000	1.000	0.000
8	1.000^h^	1.000	1.000	0.000
9	1.000^i^	1.000	1.000	0.000
10	1.000^j^	1.000	1.000	0.000
11	1.000^k^	1.000	1.000	0.000
12	1.000^l^	1.000	1.000	0.000

Statisticaly significant results are marked with bold

^a^Predictors: (Constant), Consumption_ CO_2_

^b^Predictors: (Constant), Consumption_ CO_2_, Share_of_ CO_2_

^c^Predictors: (Constant), Consumption_ CO_2_, Share_of_ CO_2_, Flaring_ CO_2_

^d^Predictors: (Constant), Consumption_ CO_2_, Share_of_ CO_2_, Flaring_ CO_2_, Other_industry_ CO_2_

^e^Predictors: (Constant), Consumption_ CO_2_, Share_of_ CO_2_, Flaring_ CO_2_, Other_industry_ CO_2_, Coal_ CO_2_

^f^Predictors: (Constant), Consumption_ CO_2_, Share_of_ CO_2_, Flaring_ CO_2_, Other_industry_ CO_2_, Coal_ CO_2_, Gas_ CO_2_

^g^Predictors: (Constant), Consumption_ CO_2_, Share_of_ CO_2_, Flaring_ CO_2_, Other_industry_ CO_2_, Coal_ CO_2_, Gas_ CO_2_, Oil_ CO_2_

^h^Predictors: (Constant), Consumption_ CO_2_, Share_of_ CO_2_, Other_industry_ CO_2_, Coal_ CO_2_, Gas_ CO_2_, Oil_ CO_2_

^i^Predictors: (Constant), Consumption_ CO_2_, Share_of_ CO_2_, Other_industry_ CO_2_, Coal_ CO_2_, Gas_ CO_2_, Oil_ CO_2_, Cement_ CO_2_

^j^Predictors: (Constant), Share_of_ CO_2_, Other_industry_ CO_2_, Coal_ CO_2_, Gas_ CO_2_, Oil_ CO_2_, Cement_ CO_2_

^k^Predictors: (Constant), Share_of_ CO_2_, Other_industry_ CO_2_, Coal_ CO_2_, Gas_ CO_2_, Oil_ CO_2_, Cement_ CO_2_, Flaring_ CO_2_

^l^Predictors: (Constant), Other_industry_ CO_2_, Coal_ CO_2_, Gas_ CO_2_, Oil_ CO_2_, Cement_ CO_2_, Flaring_ CO_2_

The results in Table 3 show that 99.99% of the decarbonisation success can be explained only by the indicator *consumption-based CO*_*2 *_ (marked with Bold numbers). By adding the indicator *share of CO*_*2*_, the model is completely explained (99.99%). All additional indicators, *CO*_*2*_* per unit of GDP* as well, that are added to the model do not contribute to further clarification of the decarbonisation success.

To further confirm that decarbonisation success cannot be explained by the indicator *CO*_*2*_* per unit of GDP*, these two indicators were added to the regression linear model as a criterion and predictor variable (Table 4.).

**Table 4:** *CO*_*2*_* unit of GDP* (a predictor variable) and *Cumulative CO*_*2*_* emission* (a criterion variable)—regression model

Model	*R*	*R* square	Adjusted *R* square	
1	0.187^a^	0.035	0.031	

^a^Predictors: (Constant), CO_2__per_GDP

The regression analysis results, in Table 4, show that *CO*_*2*_* per unit of GDP* manages to explain only 3% of the variance of *Cumulative CO*_*2*_* emission*, which is absolutely insufficient.

In the next step (Table 5), *CO*_*2*_* emission per capita* was used as a criterion variable, in order to estimate how much of variance of this dependent variable can be explained by *CO*_*2*_* per unit of GD*.

**Table 5:** *CO*_*2*_* unit of GDP* (a predictor variable) and *Cumulative CO*_*2*_* emission per capita* (a criterion variable)—regression model

Model	*R*	*R* square	Adjusted *R* square	
1	0.387^a^	0.150	0.147	

^a^Predictors: (Constant), CO_2__per_GDP

As it can be seen in Table 5, the analysis shows that only 15% of the *CO*_*2*_* per capita* emission variance can be explained by *CO*_*2*_* unit of GDP*, which is another confirmation that *CO*_*2*_* unit of GDP* should not be taken as a sufficient or even necessary indicator for drawing conclusions about the success of decarbonisation.

The analysis so far has not revealed whether the exploratory method can determine how the indicators should be grouped, in order to find a way to better define *CO*_*2*_* unit of GDP* and use it in further analysis. Therefore, the next phase of the research included the implementation of the *Principal Component Analysis*, which requires the following assumptions:

1. All variables in the model must have an interval level of measurement;
2. The linearity of the variables used in the analysis was previously confirmed through the *Pearson correlation coefficient* of the variables (Table 1).
3. Using the *Kaiser–Meyer–Olkin* test (for checking the adequacy of the sample), it was determined that the required minimum value of 0.6 was met (Table 6).
4. Using *Bartlett's test of sphericity*, it was confirmed that the data in the correlation matrix differ from zero.

**Table 6:** *Kaiser–Meyer–Olkin* test for checking the sample adequacy and *Bartlett's test of sphericity* of data

Kaiser–Meyer–Olkin measure of sampling adequacy		0.705
Bartlett’s test of sphericity	Approx. Chi-square	5402.513
	df	136
	Sig	0.000

The first insight into the values of the examined indicators is presented in the table with communalities (Table 7) which shows how much of variance of each individual parameter can be explained by the selected factors. All parameters are well represented in the selected factors and there is no need here to exclude any of these indicators (all values obtained are > 0.3).

**Table 7:** Communalities

	Initial	Extraction
CO_2_ _per_capita	1.000	0.639
CO_2_ _emission	1.000	0.994
Year_to_year_ CO_2_	1.000	0.793
Cumulative_ CO_2_ _emission	1.000	0.877
Consumption_ CO_2_	1.000	0.991
Share_of_ CO_2_	1.000	0.960
Cement_ CO_2_	1.000	0.900
Coal_ CO_2_	1.000	0.986
Flaring_ CO_2_	1.000	0.963
Gas_ CO_2_	1.000	0.978
Oil_ CO_2_	1.000	0.845
Other_industry_ CO_2_	1.000	0.969
GDP_Change	1.000	0.792
GDP_per_capita	1.000	0.881
REC	1.000	0.917
REO	1.000	0.884
CO_2_ _per_GDP	1.000	0.774

Extraction method: principal component analysis

Based on the Eigenvalues, the proposed model has 3 separate factors (the Eigenvalue of which is above 1). Auxiliary (GDP-based) indicators are also included in this phase of the research, and the results are shown in Table 8.

**Table 8:** Eigenvalues

Component	Initial eigenvalues			Extraction sums of squared loadings		
	Total	% of Variance	Cumulative %	Total	% of Variance	Cumulative %
1	11.667	68.627	68.627	11.667	68.627	68.627
2	2.237	13.161	81.788	2.237	13.161	81.788
3	1.241	7.302	89.090	1.241	7.302	89.090
4	0.732	4.306	93.396			
5	0.376	2.210	95.606			
6	0.323	1.902	97.507			
7	0.200	1.178	98.685			
8	0.127	0.749	99.434			
9	0.039	0.229	99.663			
10	0.021	0.123	99.785			
11	0.014	0.080	99.865			
12	0.011	0.062	99.928			
13	0.008	0.048	99.975			
14	0.002	0.014	99.989			
15	0.001	0.007	99.996			
16	0.001	0.004	100.000			
17			100.000			

The main goal of this analysis is to define the number of components that manage to explain most of the model variance, which would allow the monitoring model to be simplified and made more efficient and accurate. In this case, it is found that three components explain 89% of the variance of the entire model. However, it is necessary to determine which indicators overlap with them—which would indicate a methodological problem. For this purpose, factor rotation was used, by applying the *direct oblimin method* (oblique rotation, due to the determined correlation between the obtained factors) from which the *pattern matrix* is derived (Table 9).

**Table 9:** Pattern matrix with factor overlap (selected SE European countries)

	Component		
	1	2	3
Flaring_ CO_2_	1.024		
Cumulative_ CO_2_ _emission	0.991		
Gas_ CO_2_	0.988		
Consumption_ CO_2_	0.985		
Other_industry_ CO_2_	0.973		
Coal_ CO_2_	0.963		
CO_2_ _emission	0.962		
Cement_ CO_2_	0.927		
Share_of_ CO_2_	0.896		
Oil_ CO_2_	0.791		
REC	− 0.779		
CO_2_ _per_GDP	**0.657**	− **0.343**	
GDP_per_capita		0.924	
REO		0.884	
CO_2_ _per_capita		− 0.790	
GDP_Change			0.849
Year_to_year_ CO_2_			0.784

Statisticaly significant results are marked with bold

Extraction method: principal component analysis

Rotation method: oblimin with Kaiser normalization^a^

^a^Rotation converged in 4 iterations

The results in Table 9 clearly show that the principle of simple structure is almost completely satisfied, since three groups of components are defined. The first component consists of indicators related to the type of energy product or industry that is a source of *CO*_*2*_, but it should be emphasised that high overlaps exist within the first component. The second component comprises two GDP-related indicators (marked with Bold numbers), *renewable electricity output* and *CO*_*2*_* per capita.* Component no. 3 is the smallest (it contains only *GDP change* and *year-to-year CO*_*2*_) but is the most methodologically correct.

Furthermore, the indicators not contained in any other component can be obviously found in the defined three components of the model. The only, and at the same time the most problematic indicator in this model, is *CO*_*2*_* per unit of GDP*, which is in a high positive correlation with component 1 (0.657), but at the same time is in a high negative correlation with component 2 (− 0.343).

Given the performed analyses, the indicator *CO*_*2*_* per unit of GDP* is clearly insufficient to explain the overall decarbonisation, and its use, in general, can be considered questionable in the process of monitoring the decarbonisation success—it is insufficiently clearly defined, it is contained in two components of the model, and there is also a high degree of overlap with other indicators.

Considering all CO_2_-related parameters, CO_2_ emissions proved to be the most useful by type of energy product, as well as *cumulative CO*_*2*_* emissions* (which was used as a criterion variable in previous models).

### Second research stage—comparison between EU and non-EU countries

Further analysis considers differences that may exist in the values of indicators and possible model of decarbonisation in selected countries, so that EU and non-EU countries are analysed separately. To this end, pattern matrices have been developed, which show the number of components and the overlap of individual indicators. The results of this part of the analysis for the countries that joined the European Union (Estonia, Latvia, and Lithuania) are shown in Table 10.

**Table 10:** Pattern matrix with factor overlap (selected SE European countries—EU countries)

	Component			
	1	2	3	4
GDP_per_capita	− 0.977			
Share_of_ CO_2_	0.968			
REC	− 0.961			
REO	− 0.874			
CO_2_ _emission	0.796			
Cumulative_ CO_2_ _emission	0.685			
**Consumption_ CO**_**2**_	**0.660**	**0.479**		
Gas_ CO_2_		0.961		
Oil_ CO_2_		0.921		
Flaring_ CO_2_		0.833		
**Coal_ CO**_**2**_	**0.485**	− **0.766**		
**CO**_**2**_** _per_capita**	**0.485**	− **0.731**		
**CO**_**2**_** _per_GDP**	**0.622**	− **0.704**		
GDP_Change			0.842	
Year_to_year_ CO_2_			0.831	
Cement_ CO_2_				0.876
Other_industry_ CO_2_				0.398

Statisticaly significant results are marked with bold

Extraction method: principal component analysis

Rotation method: oblimin with Kaiser normalization^a,b^

^a^Rotation converged in 7 iterations

^b^Only cases for which Country_divided = EU are used in the analysis phase

Table 10 shows that a model with 4 components was proposed for EU countries, which are not clearly differentiated from each other, as is the case when the sample of all European countries was observed (Table 9). The only four factors that are methodologically correct are those related to the trend of change (*GDP change* and *year-to-year CO*_*2*_* change*), such as *Cement CO*_*2*_ and *other industries CO*_*2*_. A significant degree of overlap was detected in the case: *consumption based CO*_*2*_, *coal CO*_*2*_, *CO*_*2*_* per capita* and *CO*_*2*_* per unit of GDP*, where the correlation is positive or negative (marked with Bold numbers). All of the above calls into question the methodological correctness of the model for assessing the success of decarbonisation.

In non-EU countries (Russian Federation, Moldova, Armenia, Azerbaijan, Georgia and Ukraine), the model has the least methodological objections than in EU countries. The pattern matrix is shown in Table 11.

**Table 11:** Pattern matrix with factor overlap (selected SE European countries—non-EU countries)

	Component		
	1	2	3
Flaring_CO_2_	0.987		
Other_industry_CO_2_	0.958		
Cement_CO_2_	0.949		
GDP_per_capita	0.921		
Consumption_CO_2_	0.870		
CO_2_ _per_capita	0.865		
Coal_ CO_2_	0.830		
Oil_ CO_2_	0.817		
CO2_emission	0.806		
**Share_of_CO**_**2**_	**0.661**	− **0.510**	
REO	− 0.604		
REC		0.965	
CO_2_ _per_GDP		− 0.930	
Cumulative_CO_2_ _emission		0.815	
**Gas_CO**_**2**_	**0.605**	− **0.672**	
GDP_Change			0.978
Year_to_year_CO_2_			0.961

Statisticaly significant results are marked with bold

Extraction method: principal component analysis

Rotation method: oblimin with Kaiser normalization^a,b^

^a^Rotation converged in 14 iterations

^b^Only cases for which Country_divided = Non_EU are used in the analysis phase

Table 11 shows that in this case, as well, there are factors that are problematic—*Share of CO*_*2*_ and *Gas CO*_*2*_ (marked woth Bold numbers). In these countries, it is more correct to use *CO*_*2*_* per unit of GDP* to assess the success of decarbonisation, as it is an indicator that is clearly present in the second component, without overlapping with others, together with *renewable energy consumption* and *cumulative CO*_*2*_* emission*.

## Discussion, recommendations and future sustainability concerns

As a final conclusion, after trying to optimise the model with a tendency to group factors into CO_2_-related and GDP-related, as well as those that relate to a trend of change, it is clear that such measurement of decarbonisation is not possible, i.e., it is methodologically incorrect. It is proposed to use either measures that are based exclusively on CO_2_ (such as CO_2_ emissions by different industries) or measures that have a determinant of GDP, which is less useful in this model. The indicator *CO*_*2*_* per unit of GDP* is a poor attempt to reconcile these measures, as being obviously not clearly defined and therefore certainly making a modest contribution to the explanation of this model, i.e. not being able to show the decarbonisation success. An additional problem is that importance of certain indicators is different in different countries (EU or non-EU); therefore, this should be kept in mind when creating a universal model.

Monitoring based on a single model (to be implemented by the EU) must be country-specific. Otherwise, some countries will be at a disadvantage as monitoring might show they are not successful enough in the decarbonisation process. On the other hand, a methodologically incorrect model may show that decarbonisation in some countries is carried out extremely successfully. In both cases, countries would not have the same treatment and would bear the long-term consequences of their failure/success in the decarbonisation process, while decisions on further decarbonisation activities (and spending large funds) would be made on the wrong monitoring results.

In addition to the above shortcomings, it should be borne in mind that the existing ways of measuring the success of decarbonisation (as well as the ways of fighting climate change in general) do not take into account indicators that are of particular importance for stable and sustainable development. Given the ambitious plans of the European Union in the field of decarbonisation, the issue of adequate monitoring is becoming increasingly pressing.

The European Union has further intensified its commitment to climate neutrality in its strategic *Post-COVID-19 Recovery Plan* [40]. This plan, which envisions the largest budget adopted by the European Union in its history, sets out priority areas for Europe's development by 2050: climate change, digital transition, resilience, security, health improvement and education. These areas are individually complex, but interrelated and mutually connected by impacts that have to be studied, predicted and monitored as well, with the aim of gaining a realistic picture of trends that will cover the entire Europe by 2050, with numerous (currently remaining unclearly defined) consequences among which social, economic and security ones stand out as particularly sensitive.

All activities on decarbonisation have (and will have in the future) a great impact on the economic development of Europe, the well-being of its citizens, relations with neighbouring countries, as well as on global trends as a whole. Therefore, decarbonisation should not be appraised as a separate and one-way process (with activities that encourage the process), but has to be carefully considered, and the indicators that are not currently used have to be included in the monitoring process. The research conducted for the purpose of this paper has shown that there is no clear monitoring model, that the indicators currently used are often ineffective (or result in misleading information), and that only the status of environmental parameters are considered, which is by no means adequate. Using GDP in monitoring models cannot be considered sufficient because the economic implications completely exceed this (often disputed) macroeconomic indicator. The social welfare and security aspects are completely neglected, and are directly related to decarbonisation process, with the expected long-term effects.

The European Union endeavours to base its future economy on the principle of circularity, with as little resource consumption and as little waste as possible. In addition, Europe is giving a strong impetus to electrification, with the aim of decreasing the use of fossil fuels. The foregoing is possible by producing energy from certain renewable sources, but it covers only a small part of energy needs. Moreover, renewable energy production has proven to be an expensive investment for a large number of countries, therefore numerous assessments and reviews are being conducted of the sustainability of renewable energy production in all aspects.

For that reason, a need for further imports of natural gas (as an environmentally friendly energy source), primarily from the Russian Federation, became an imperative of decarbonisation and electrification. In this respect, after the commissioning of Nord Stream 1 (2011), the European Union predicted that the need for natural gas will increase. Thus, the works on Nord Stream 2, intended to supply Germany, but also other countries of the European Union, began in 2017 and were completed in 2021. Activities on the implementation of Nord Stream 2 have caused much turmoil on the international political scene, with the emergence of security problems and military activities in the countries of the European Union that border the Russian Federation. In this way, decarbonisation and energy consumption are becoming a very important security issue, with the potential to create crisis situations of different types and coverage.

The general opinion that decarbonisation has a positive impact on the environment and the general benefit of society can be true in the long run. However, the total costs of energy transition and their impact on the economy of each country must be considered in order to achieve this long-term goal. It is essential to make agreements on fair distribution of burdens, both at the international level, between rich and poor countries, and between EU and non-EU countries, because they do not have access to EU funds to support energy transition. The cost of transition is also affected by the speed of its implementation. Slowing down the energy transition can reduce losses in terms of premature capital obsolescence, facilitate the transfer of resources from consumption to investment, and allow technological advances to reach better and more efficient solutions. This would provide time to reach some more just solutions on the redistribution of income and wealth (regardless of the energy transition) given a growing trend of social inequality, which is not primarily caused by energy transition. In conditions of growing inequality, energy transition further fuels differences and becomes politically unsustainable given damaged credibility of international institutions and a lack of interest in a globally coordinated approach, which is essential for the success of global activities.

However, at the same time there is a so-called carbon spill over, because EU countries are entitled to free allowance to prevent pollution from heavy industry, under the pretext of trying to stop its relocation outside of Europe. Moreover, Members of the European Parliament voted in March 2021 that free allowance should remain in use even when the planned cross-border adjustment mechanism is in place. Therefore, it is obvious that the interests of the EU member states are protected in this way. European non-EU countries may be the target of criticism for poor results in decarbonisation, where the methodological correctness of monitoring is not being questioned by the European Commission.

In line with the ambitious goal of EU countries to reduce CO_2_ emissions by 2030 by 50–55% compared to 1990, the *Carbon Border Adjustment Mechanism* (CBAM) was adopted in July 2021, which implies a tax on imports of certain products from third countries that do not have the same ambitions in terms of decarbonisation. The rationale for introducing this mechanism is that decarbonisation cannot be achieved to that extent if EU production moves to third countries, which have more lenient emission regulations. The idea is to support the decarbonisation process through the taxation of imports of products, in the production of which significant amounts of CO_2_ are emitted, and to use the generated income to support the goals from the European Green Deal. However, the question arises as to whether this mechanism is an example of a protectionist measure, which could be strengthened by problematic monitoring.

The proposed CBAM mechanism implies the introduction of taxes on the import of all products and raw materials covered by the EU Emissions Trading System (EU ETS) and taxes to be imposed by 2023 on the electricity sector and industry that consumes a lot of energy (manufacture of cement, steel, aluminium, paper, glass, chemicals and fertilizers).

Defining an adequate structure of the economy and attracting investments is the basis of a long-term development strategy, which includes synchronised structural reforms not only in the industrial, energy and environmental sectors, but also in other sectors, which should contribute to creating resources and infrastructure for a decades-long turnaround. For small open economies of European non-EU countries, this measure and similar ones imply prioritising the development of innovation-based economy, but also creating a flexible and diplomatic approach, in order to adapt the transition process to regional specifics. Proper monitoring is of great importance for all of the above mentioned.

Energy transition will certainly affect the labour market, not only in terms of job losses in the energy sector but also in energy-intensive industries, which will require special support and retraining programmes. In fact, there are about 18 million employees in the energy industry worldwide, and it is estimated that there will be 26 million by 2050, but on the other hand, it has been argued that decarbonisation is a jobs killer. About 12.6 million people are employed in the energy sector worldwide, 4.6 million in the renewable energy industry and 0.8 million in the nuclear sector. Decarbonisation will actually enable [41] that of total energy jobs in 2050, 84% would be in the renewables sector, 11% in fossil fuels, and 5% in nuclear. Therefore, by 2050, the UK Government’s goal is 80% decarbonisation, while at the same time improving productivity, as well as creating new employment opportunities [42]. In the EU, between 2020 and 2050, almost half of the decline will be in low-skilled jobs, a 25% drop in middle-skilled jobs and a 50% increase in high-skilled jobs. It is projected that for every additional million euros worth of batteries produced in the Union, 3 full-time equivalent jobs will be created, and also around 140,000 new jobs in the renewable energy sector will be made by decarbonisation effects by 2050 [43].

The EU will undoubtedly facilitate the transition of its member states through the JTC and other mechanisms and funds (e.g., *European Regional Development Fund* and *European Social Fund Plus*), but third countries can neither count on the same funds nor have a technologically competitive industry. Additional taxation will negatively affect price competitiveness and create pressure to invest in advanced technologies, but without systemic support mechanisms, private capital seeks channels and a more stimulating business environment, with a widening gap between rich and poor.

The countries of Eastern Europe (former members of the USSR) are in a special position on several grounds. They can provide decarbonisation and intensive electrification primarily by increased gas imports from the Russian Federation, with minimal opportunities for diversification from other sources. On the other hand, the countries of Eastern Europe, which are at the same time members of the European Union, are additionally at risk due to their membership in the NATO alliance.

Given the aforementioned observations, further monitoring of the success of decarbonisation (as well as the models thereof) should take into account the selected social and security parameters, while expecting to develop models to enable quantification of indicators that are currently not quantified or expressed in unit of value, in standard manner.

## Conclusions

The main goal of this paper is to assess the methodological correctness of certain combinations of indicators for monitoring the decarbonisation success in the EU countries, used around the world. The aim of this paper is selected taking into account that the carbon emission reduction is considered one of the most important elements of global climate policy, with the European Union committed to achieving climate neutrality, i.e. net-zero carbon emission by 2050. Such an ambitious goal, strategically defined in *European Green Deal* (2018), legally regulated in *European Climate Law* (2021) and operationalised in *European Recovery Plan* (2021), requires the adequate methods of planning and monitoring the success of implementation, which has not been specified so far. Decarbonisation is a special challenge in other European countries and in the Russian Federation (energy-intensive technologies in use, low energy efficiency, moderate investments in renewable energy sources and high carbon emissions), where monitoring the success of energy transition is of great importance.

Carbon-related monitoring is largely performed (by monitoring certain indicators), but adequate monitoring that will support the implementation of the plan on European climate neutrality by 2050 is difficult and problematic on several grounds. First of all, the monitoring system is not clearly defined, despite the fact that the reduction of carbon emissions is widely monitored, and that an ambitious budget is provided for the implementation of climate neutrality. There is only a specified plan to develop a monitoring system, but without any details in that direction. This imposes the continued use of existing indicators that indicate the status of decarbonisation, but their use and conclusions drawn on this basis are questionable. In fact, the number of indicators is large, many of them are methodologically vaguely defined, their correlations have not been sufficiently examined, and the data collection system is problematic—which calls into question their reliability.

The analysis conducted in this paper indicates the basic methodological characteristics of the existing approach to measuring the decarbonisation success. A clear approach is not defined by the competent bodies of the European Union, so the indicators used by *Eurostat* and the *International Energy Agency*, which clearly refer to CO_2_, are used for the analysis.

Methodological correctness was checked by using adequate statistical methods, and the basic analysis result indicates important problems in monitoring. First of all, monitoring should be based on the use of a significantly smaller number of indicators than is currently the case. The analysis showed that monitoring using only three or four indicators (out of 17 included in the analysis) would be very successful, with differences between EU and non-EU countries. The indicator *consumption-based CO*_*2*_* emission* and *share in global CO*_*2*_ stands out as the most useful in EU countries. On the other hand, in non-EU countries, *CO*_*2*_* per unit of GDP*, *consumption-based CO*_*2*_, and *renewable energy consumption* proved to be the most efficient indicators. The analysis showed that the indicator *CO*_*2*_* per unit of GDP* should not be used in the EU countries. Moreover, monitoring the share of energy consumption in EU countries does not significantly contribute to the success of monitoring, and its use for this purpose should be considered. The indicator *year-to-year CO*_*2*_* emission change* proved to be reliable for both groups of countries, so its use is recommended, but best as a stand-alone indicator. For non-EU countries, it is recommended to group the indicators into three groups: indicators that show CO_2_ sources, indicators based on GDP and indicators of change. Joint observation of all three groups of indicators is by no means recommended, as the analysis showed a high degree of unreliability in such case.

Given the research results, as well as the existing issues in defining the decarbonisation monitoring, further studies should be focused on creating a country-specific model of decarbonisation. Consequently, the issues that inevitably arise from incorrect monitoring results will be avoided: poor decision-making, poor long-term plans, unreliable investments, but also legal consequences that may result from the fact that a country shows poor results in terms of decarbonisation. In this way, a more efficient realisation of the common goal of decarbonisation will be supported, while contributing to the reduction of evident disagreements among EU countries (and neighbouring countries), when it comes to energy and climate policies.

Certainly, further research must include the social, economic and security aspects of decarbonisation. Namely, the transition to environmentally friendly energy sources (especially electrification and use of natural gas—currently primarily from the Russian Federation) in the region of Eastern Europe leads to significant changes in all spheres of society, which can have significant implications for the development of each country in this region and Europe as a whole.

## Data Availability

The datasets used and/or analysed during the current study are available from the corresponding author on reasonable request.
